# Emerging trends in nano-sensors: A new frontier in food safety and quality assurance

**DOI:** 10.1016/j.heliyon.2024.e41181

**Published:** 2024-12-12

**Authors:** Farhang Hameed Awlqadr, Ammar B. Altemimi, Syamand Ahmed Qadir, Tablo Azad Hama Salih, Zina T. Alkanan, Qausar Hamed AlKaisy, Othman Abdulrahman Mohammed, Mohammad Ali Hesarinejad

**Affiliations:** a Food Science and Quality control, Halabja Technical College of Applied Science, Sulaimani Polytechnic University, Sulaymaniyah, Iraq; b Food Science Department, College of Agriculture, University of Basrah, 61004, Iraq; c College of Medicine, University of Warith Al-Anbiyaa, Karbala, Iraq; d Medical Laboratory Techniques Department, Halabja Technical Institute, Research center/Sulaimani Polytechnic University, Sulaymaniyah, Iraq; e Department of Dairy Science and technology, College of Food science, Al-Qasim Green University, Iraq; f Medical Laboratory Science Department, Halabja Technical College of Applied Sciences, Sulaimani Polytechnic, Iraq; g Department of Food Sensory and Cognitive Science, Research Institute of Food Science and Technology (RIFST), Mashhad, Iran

**Keywords:** Bacteria, Biosensors, Foodborne, Nanotechnology, Nano-sensor, Pathogenic

## Abstract

The rapid evolution of nanotechnology has catalyzed significant advancements in the design and application of nano-sensors, particularly within the food industry, where ensuring safety and quality is of paramount concern. This review explores the multifaceted role of nano-sensors constructed from diverse nanomaterials in detecting foodborne pathogens and toxins, offering a comprehensive analysis of their operational principles, sensitivity, and specificity. Nano-sensors leverage unique physical and chemical properties at the nanoscale to enhance the detection of microbial contamination, actively contributing to food safety protocols. With applications ranging from real-time monitoring of pathogenic bacteria, such as Escherichia coli and Salmonella, to assessing environmental factors affecting food quality, these innovative devices demonstrate unparalleled advantages over conventional detection methods. Recent research illustrates the integration of nano-sensors with biosensing techniques, enabling multiplex analysis and rapid detection. Furthermore, the review addresses current challenges in the commercialization and regulatory landscape of nano-sensor technology, emphasizing the need for ongoing research to optimize their performance and facilitate widespread adoption in food safety systems. Overall, the incorporation of nano-sensors represents a transformative approach to safeguarding public health by proactively managing food safety risks and enhancing the efficiency of food quality assurance processes.

## Highlights

• Nanotechnology enhances food safety and shelf life by detecting and monitoring microbial contamination.
• Nano sensors can detect pathogens like *E. coli* and *Salmonella* with exceptional sensitivity.
• Nano sensors are categorized as physical, chemical, or biological, each serving specific detection purposes.
• Various nanomaterials and manufacturing techniques enable the creation of highly sensitive nano sensors.
• Nano sensors play a crucial role in detecting various foodborne pathogens with exceptional sensitivity and specificity.

## 1 Introduction

The field of nanodevices includes the study of their manipulation, development, application, and analysis techniques, as well as their operating modes. Nanotechnology is used extensively in producing nanocomposites and nano-sensors [1]. The nanomaterials used have diverse chemical, physical, and surface properties, but are limited in size to 100 nm. In the food sector, nanotechnology is applied for the development of nano sensors and nanoscale food products. Nanomaterials help improve the shelf life of food products by removing gas and moisture. Nano sensors are used for assessing the safety and quality of food during preparation and packaging. Nanotechnology has the potential to address concerns related to food safety, food processes, and food packaging [2]. Nanotechnology in food packaging is particularly interesting as it enhances the quality and safety of food products. Nanotechnology offers several advantages to the food packaging industry, including the ability to detect microbial contamination. Nano sensors have become a valuable tool in the food industry [3,4].

Nano sensors convert physical or chemical quantities into easily detectable and analyzable signals. They are used to detect pesticides, food spoilage, toxins, foodborne pathogens, and undesirable tastes or smells. They are also used to monitor time, temperature, and oxygen levels [5]. Additionally, nano sensors play a crucial role in detecting foodborne pathogenic bacteria. The incidence of foodborne illnesses caused by contaminated products is increasing globally. Ingesting food tainted with bacteria, viruses, and parasites has been linked to approximately 250 separate illnesses [6]. Managing these interconnected illnesses places a significant burden on healthcare and socio-economic stability. According to the European Food Safety Authority (EFSA), the number of foodborne outbreaks, cases, hospitalizations, and deaths increased in 2022 compared to the previous year, with the main causes being *L. monocytogenes* and *Salmonella* [7]. Although most bacterial strains are benign, a subset of them possess pathogenic properties. Foodborne pathogens, such as (*Campylobacter jejuni, Escherichia coli* type O157H7, *salmonella subspecies*, *Clostridium perfringens*, *Vibrio species*, *Shigella species*, *Listeria monocytogenes*, and *Clostridium botulinum)*, are the primary causes of disease outbreaks [8,9].

Regular examination for microbial contamination is essential to guarantee the quality and safety of food. Nanomaterials have been recently incorporated into biosensing systems to enable multiplex analysis of foodborne bacteria. This integration offers improved sensitivity and reduced detection time. Nanomaterials are typically combined with biomolecules such as enzyme, antibody, and nucleic acid in sensor applications to achieve the desired specificity. Nano sensors for foodborne pathogens and toxins have been created by utilizing the optical and electronic characteristics of nanomaterials [10].

The target or food compound binds to organic molecules, resulting in the formation of detectable outputs through biological signals. These signals are identified by a transformer, which can detect signals such as (electro-chemical, optical, electric, calorimetric, acoustic, and mechanical). The sensor's design depends on the reaction between a biological element and substratum. The development of nano sensors involves the utilization of confinement and fabrication techniques to integrate nanomaterials and nanoparticles with transducers. The nanotechnology recent researches are focusing on the development of nano sensors, which possess significant potential because of their high-rise sensitivity, small size, quick response, and streamlined research techniques. Additionally, biosensors offer the ability to perform bioanalytical methods [11].

The review focuses on recent advancements and concerns related to nano sensors based on nanomaterials. These sensors have shown significant potential in detecting foodborne pathogens with exceptional sensitivity and specificity, while also offering advantages such as reduced reagent volume and shorter detection times, thus improving food safety.

## 2 Nano-sensor

### 2.1 Definition

Nano-sensors are constructed using materials at the nanoscale level (10^−9^ m) or have the ability to detect material or food components at the nano range level (10^−9^ m) [12]. Nano-biosensors are analytical sensor devices capable of detecting biological agents at the nano range. These sensors are constructed using materials that are at least 1 nm in size to detect biological agents [13]. The quantification and detection of target pathogens have been achieved by utilizing the biochemical reactions triggered by the binding of bio-receptors such as enzymes, antibodies, and nucleic acid. When the bioreceptor forms a strong connection with the target pathogen, it induces alterations in optical, thermal, or electrical characteristics, which can be detected using biosensors [14]. Nano sensors can be categorized into three main types based on the signal they generate while interacting between the bio receptor and the pathogen, as well as their construction. The most exemplary types of nanoparticle-based nano sensors are those that utilize magnetic nanoparticles (MNPs), nanoparticles made of metals such as gold (Au) or silver (Ag), and silica nanoparticles [15]. These nano sensors utilize a sensor fabrication material at the nanoscale level. Optical nano sensors generate optical signals, while electrochemical nano sensors produce electrical signals when there is binding between bio receptors and target analytes.

### 2.2 Nano-sensors categorization

**Fig. 1:**
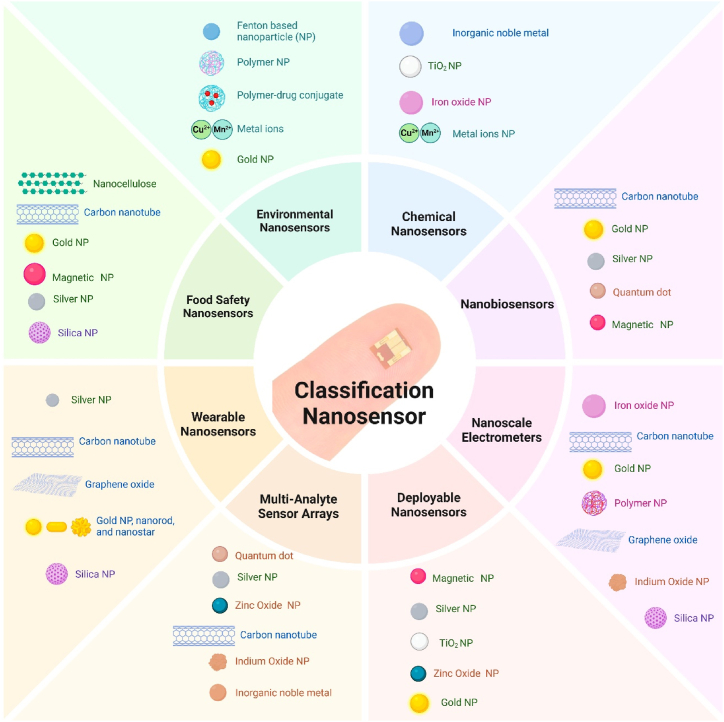
Functional Classification of nanosensors.

Nano-sensors can be classified into three categories according to the type of signal they detect: physical nano-sensors, chemical nano-sensors, and biological nano-sensors. Furthermore, they can be further categorized based on their applications, framework, and source of energy. Fig. 1 provides a description for certain nano-sensors, enumerating different types. Mechanical nano-sensors have the capability to precisely quantify stress, strain, and pressure, making them crucial elements in the functioning of microelectronic nanodevices. They frequently exhibit superior performance in terms of accuracy and dependability when compared to optical and electromagnetic counterparts. The fluidic shear stress carbon nanotube and nanomechanical cantilever sensors are the most sophisticated options available. For instance, a specific research conducted an engineering nano-sensor to quantify the oscillation and elasticity of nanospheres [16].

Chemical nano-sensors, including gas nano-sensors, are utilized in diverse fields such as medical, ecological surveillance, food quality and safety, and national security. Pearton et al. have discovered chemical sensors that possess the capacity to analyze a broad range of environmental and biological gases and liquids, demonstrating their versatility. These sensors demonstrate exceptional sensitivity and have the ability to discern and detect specific analytes with precision [17]. Gas sensors, for example, measure gas volume by undergoing oxidation or reduction reactions and quantifying the resulting change in electric current. A sensor composed of zinc oxide (ZnO) nanobelts has the ability to accurately detect ammonia (NH_3_) [18].

Optical nano-sensors are utilized to observe and analyze the chemical makeup of the surroundings. These sensors facilitate meticulous chemical analysis by quantifying the optical characteristics of materials. Optical nano-sensors find applications in the domains of biotechnology, environmental science, and chemistry. They utilize photovoltaics and a single binding component. Luminescence is frequently involved in their operation, as it arises from the absorption of light by a fluorophore followed by its subsequent emission [19]. The initial optical nano-sensor, which utilized fluorescein, was employed specifically for pH measurement. Vo-Dinh et al. have conducted research on the innovative application of fiber optic sensors for highly effective in vivo monitoring of cells. The reaction between a target and receptor gives rise to physiochemical phenomena, which are subsequently converted into a measurable electrical signal [20].

**Fig. 2:**
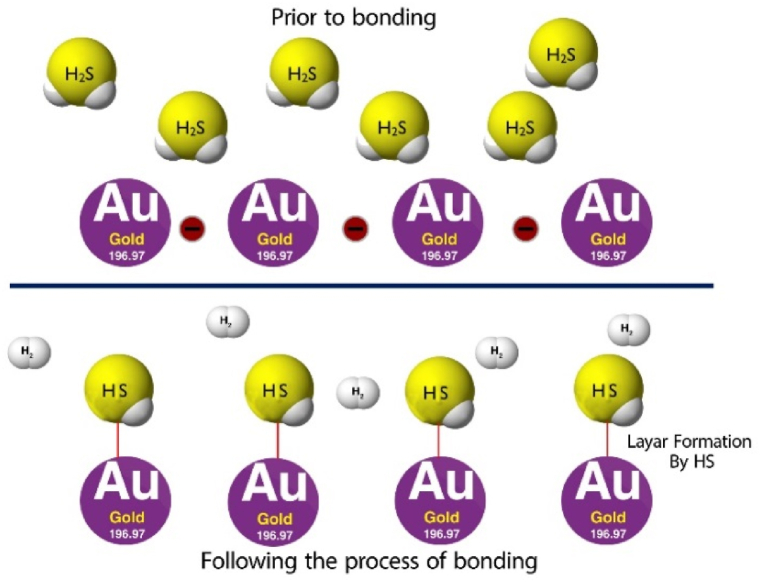
Electron migration prior to bonding and following the process of bonding.

In the study examined the reaction between AuNPs and H_2_S gas molecule [21]. They observed that the formation of a sulfide shell promotes the transfer of charge between two nanoparticles, a phenomenon referred to as hopping. This is depicted in Fig. 2. In addition, magnetic nano-sensors, which comprise of magnetic nanoparticles such as iron oxide, engage with particular substances to create long-lasting nano assemblies. This interaction can lead to a modification of the spin–spin relaxation time, a phenomenon that can be observed through Magnetic resonance imaging (MRI) and is frequently working in the detection of biomolecules [22].

**Fig. 3:**
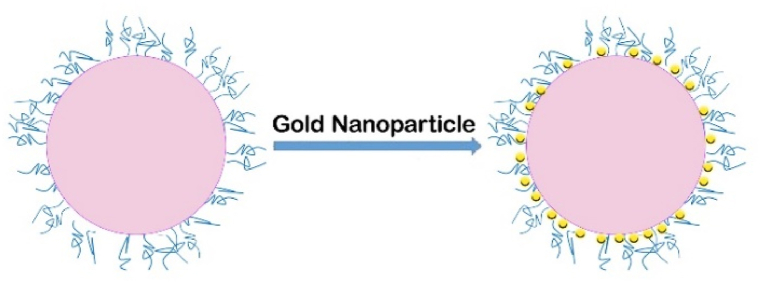
Casein-coated gold nanoparticles. Proteases degrade the protective casein barrier, thereby exposing the surface of the gold nanoparticles.

**Fig. 4:**
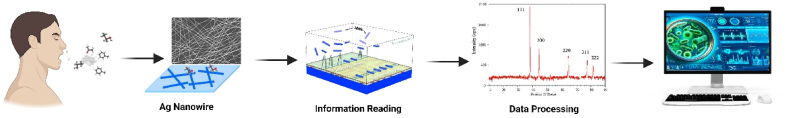
Detect VOC present in breath released.

Biological nano-sensors, a type of chemical nano-sensor, have the ability to simultaneously detect entities such as cancer, specific DNA, and different diseases. Field-effect transistors are highly efficient biosensors as a result of their ability to be produced in large quantities, their inexpensive, and their exceptional sensitivity in detecting substances. The combination of biosensor and microfluidic technologies on chips has demonstrated durability and efficiency within the body, leading to their widespread use in diagnosis and signaling a novel approach to biosensing that merges chemical and biological elements [23]. Furthermore, optical nano-sensors commonly employ noble metal and metal oxide nanoparticles for material categorization. In addition to nanoparticles, semiconductor quantum dots (QDs) are utilized for the creation of sophisticated nano-sensors. In certain nano-sensors, nanoprobes containing dye are employed to suppress fluorescence when an analyte is present. A specific instance is a biomarker composed of Au nanoparticles that can identify protease diseases in humans [24]. Fig. 3 illustrates the process of detecting these proteases. Carbon nanotubes (CNTs) possess high strength and lack reactivity, making them well-suited for chemical doping and functionalization. This enhances their capacity to selectively identify specific target analytes. A significant number of carbon nanotube (CNT) nano-sensors are designed as field-effect transistors. Chemo-resistive sensors composed of nanofibers and nanowires are highly skilled at diagnosing diseases [25]. They have the ability to detect volatile organic compounds present in breath released, as shown in Fig. 4.

Graphene nano-sensors are employed in biomedical and chemical sensing applications to identify particular gases by analyzing changes in the noise spectra of the graphene transistors. The utilization of high-conductivity three-dimensional graphene sheets has been observed in diverse industries, while graphene foams exhibit numerous promising applications [26]. Bulk nanostructured sensors offer certain advantages in certain cases. The nanoparticles' great range surface area enables them to coat biomaterials and immobilize molecules. Electrochemical nano-sensors derive advantages from the catalytic properties of nanoparticles, specifically platinum (Pt) nanoparticles combined with porous carbon, which exhibit exceptional effectiveness in gas diffusion electrodes [27]. Metal-organic frameworks (MOFs) are porous substances composed of cations of metallic elements and organic molecules known as "linkers". They are extensively utilized for gas sensing purposes due to their substantial surface area and hollow structure [28].

### 2.3 Current limitations of nano-sensors

The limitations of nano-sensors present significant challenges for their widespread adoption across industries, including food safety, healthcare, and environmental monitoring. High production and operational costs stand out among the major hurdles. Advanced materials such as noble metals, essential for high-performance sensors, require expensive fabrication methods like chemical vapor deposition and lithography, limiting scalability [29]. Furthermore, nano-sensors are highly susceptible to environmental conditions despite their promising sensitivity and selectivity. Temperature, humidity, or light exposure changes can degrade performance, impacting real-world applications [30].Another challenge involves scalability and standardization. As nano-sensors' production transitions from laboratory prototypes to industrial-scale manufacturing, ensuring consistency and reproducibility across batches remains difficult. Variability in material properties such as size and surface chemistry hampers standardization efforts, limiting broader commercialization [31]. The regulatory landscape further complicates the adoption of nano-sensors. As nanomaterials introduce uncertainties regarding long-term environmental and health impacts, regulatory bodies are cautious in approving these technologies, especially for food safety applications. This creates uncertainty for companies, slowing down investments and market adoption [32]. However, their efficient speed has led some companies to produce and put it at the service of society. Table 1 shows the types, manufacturers and characteristics. Fabrication methods also represent a bottleneck. Techniques like lithography and chemical etching are complex, time-consuming, and prone to defects. Achieving precision in sensor production at scale is difficult, requiring more efficient, cost-effective manufacturing methods [33]. Moreover, the lack of high-resolution sensor images and the need for innovative packaging solutions for lab-on-chip technologies underscore further challenges in design and integration [34].

**Table 1:** Types, manufacturers, and characteristics of nano-sensors used in food safety.

Type of Nano-Sensor	Manufacturer	Characteristics	Use Case
Optical Nano-Sensor	Tellspec Inc.	Provides rapid, non-invasive detection of food contaminants and allergens	Detection of allergens in packaged foods
Electrochemical Nano-Sensor	Biosensia Ltd.	Measures chemical and biological analytes with high sensitivity and specificity	Monitoring freshness and spoilage in perishable goods
Magnetic Nano Sensor	MagID	Utilizes magnetic nanoparticles to detect pathogens with minimal sample preparation	Identification of bacterial contamination in food samples
Carbon Nanotube Sensor	Nanomix Inc.	Highly sensitive and selective detection for gases and volatile organic compounds	Detection of spoilage gases in food packaging
Plasmonic Nano-Sensor	Plasmore Srl	Employs plasmonic resonance for enhanced sensitivity to chemical and biological substances	Detection of toxins and chemical residues
Lab-on-a-Chip Nano-Sensor	Microfluidic ChipShop	Integrates multiple sensors on a microchip for real-time analysis of food quality	Comprehensive testing for foodborne pathogens at point-of-care
Biosensing Nano-Coatings	NanoBioMatters	Enhances packaging surfaces to detect spoilage or harmful bacteria	Real-time spoilage detection in food packaging
Quantum Dot Nano-Sensor	QD Vision	Emits light signals for fast and highly accurate detection of contaminants	Detection of heavy metals in food and beverages
Nanopore Sensor	Oxford Nanopore	Performs single-molecule analysis of nucleic acids and proteins for microbial detection	Detection of pathogens through DNA sequencing

### 2.4 Manufacturing of nano-sensors

**Fig. 5:**
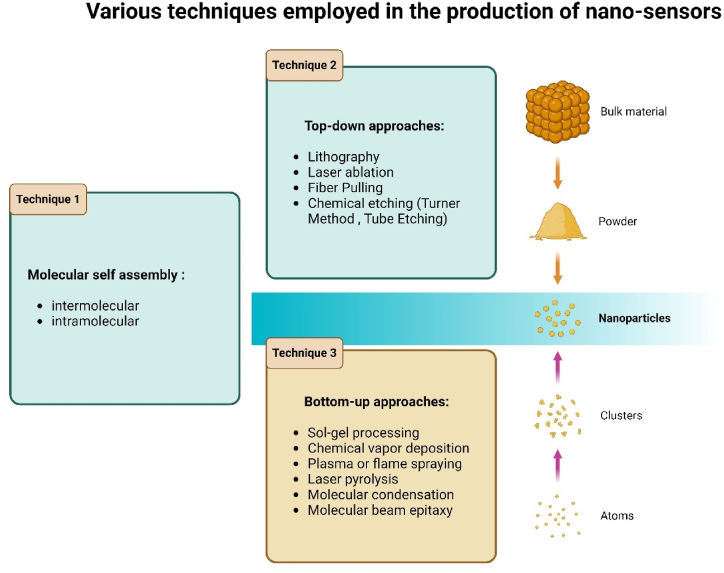
The key differentiation between top-down and bottom-up methodologies.

Nano-sensors are manufactured using various techniques, with the most prevalent methods being top-down, bottom-up utilization, and self-assembled nano-structure. The fundamental distinction between the top-down and bottom-up methods is illustrated in Fig. 5 and depicts various techniques employed in nano-sensor production.

#### 2.4.1 Methods top-down

The top-down method, common in microelectronics, involves creating nanoscale shapes by subtracting material from the original deposited material. This method can produce items a few tens of nanometers in size and is tightly controlled to ensure precise size and shape. The top-down nano-sensor production methods are described below [35].

##### 2.4.1.1 Lithography

**Fig. 6:**
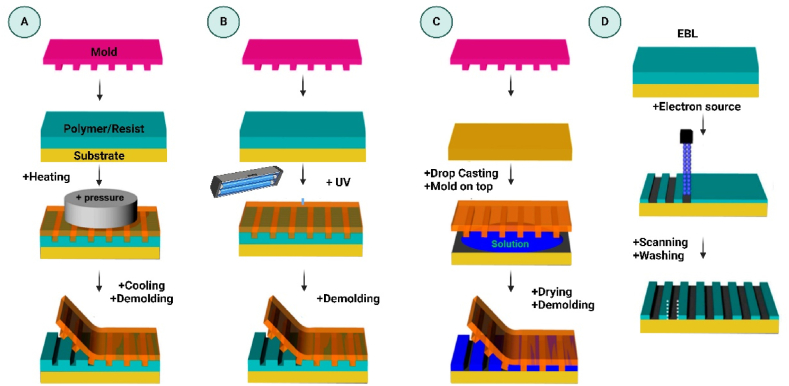
(A–C) Nanoimprint lithography (NIL) examples: thermal (TNIL), ultraviolet (UVNIL), and moulding in capillaries (MCNIL) (D) Electron-beam lithography (EBL).

Nano-sensors made via lithography can identify pollutants and quality markers at the molecular level, improving food safety. Nanoimprint lithography (NIL) Fig. 6(A–D) shows some types of fabrications Nanoimprint, soft lithography, and electron-beam lithography (EBL) all have advantages for generating sensitive and specialised sensors [35]. NIL combines pressure and heat to transfer nanoscale features onto a polymer layer using a mold, allowing for high-fidelity, low-cost structures. Paper-based plasmonic sensors that detect biogenic amines, food deterioration markers, use reversal nanoimprint lithography, a form of NIL. Nanoparticles are embedded onto flexible substrates. Because they are cheap, flexible, and eco-friendly, these sensors are perfect for disposable food monitoring systems [36]. Moreover, Soft-lithography Popular soft lithography creates reusable sensors that send real-time data, including NH₃ concentrations in food packaging, to cellphones for easy monitoring. The technology is suitable for rapid food safety detection due to its low cost and adaptability for flexible substrates [37]. In addition, Electron-beam lithography produces nanoscale designs with sub-10 nm feature sizes with unmatched precision EBL is essential for sensors that detect extremely low microbial or chemical contamination, but it is more expensive and time-consuming than NIL or soft lithography. Food safety biosensors and nanosensors with great specificity and stability are made with EBL, but its cost limits large-scale manufacture [38]. Finally, Food Safety Nano-Sensor Applications Lithographic technologies have enabled the development of various nano-sensors for food safety monitoring. NIL-based nanopillar arrays improve cell adhesion, allowing food samples to be tested for bacterial infections, reducing foodborne diseases [39]. NIL and EBL can also make sensitive polymer-based nanostructures that can detect chemical residues and pollutants in food, providing cost-effective and scalable food monitoring and safety solutions [40].

##### 2.4.1.2 Pulling fiber

**Fig. 7:**
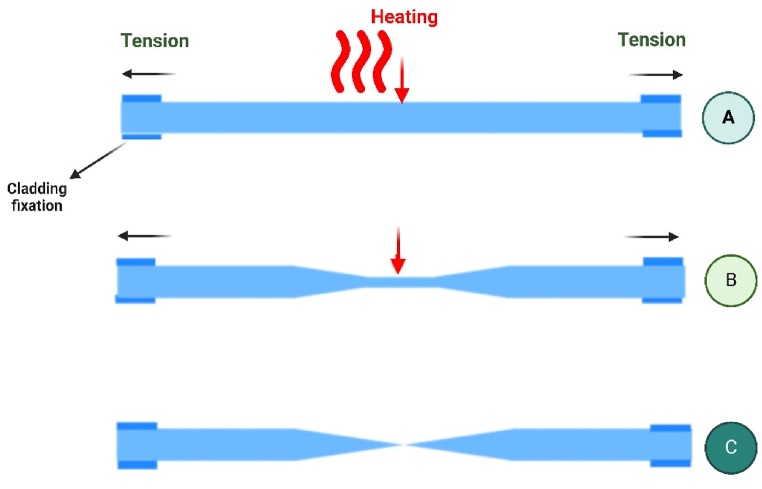
Schematic representation of the thermal pulling process (A). An input fibre component. Biconical taper featuring a central waist (B). Two optical fibre tips(C).

The pulling fiber method, commonly utilized in the field of technology in fiber optics, involves exerting a tensile force on a fiber to achieve nanoscale dimensions along its main axis. To facilitate elongation, the fiber is heated [35]. Fig. 7 graphically illustrates the thermal pulling process. Due to their great sensitivity and particular surface contacts, pulling fibre nano-sensors may detect pollutants, pathogens, and chemical residues at low concentrations. In particular, fibre optic-based sensors with nanofiber tapers may detect volatile organic compounds (VOCs), which indicate food spoilage. These nanofibers are ideal for real-time food quality monitoring due to their great transmission efficiency and sensitivity. Combining these fibres with functional coatings like metal oxides or carbon nanotubes can increase selectivity for certain analytes like ammonia in packaged goods, making them more useful for food freshness monitoring and spoiling prevention [41].

##### 2.4.1.3 Chemical etching

**Fig. 8:**
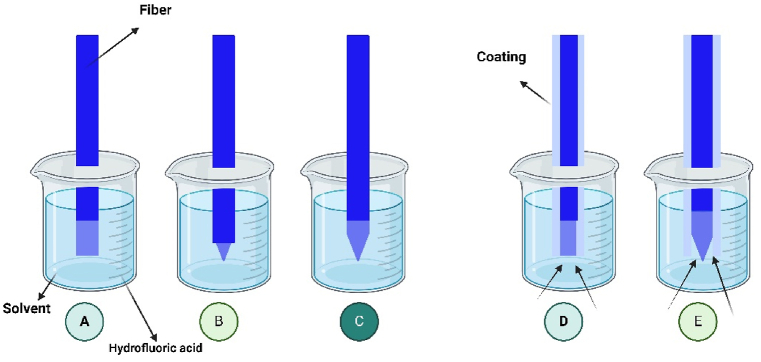
Diagram of Turner etching. (A)Start of etching. (B) Etching is still in progress. (C)Etching ending. Then the tube etching technique. (D)Start of the process. (E)Intermediate process step, such as the creation of the tip.

The main chemical etching methods for nano-sensor fabrication include the Turner method and Tube etching. Tube etching utilizes hydrofluoric acid to corrode the optical fiber that is inserted within. This technique involves submerging the silica fiber, coated with an outer protective material, in hydrofluoric acid, which specifically corrodes the end of the fiber while leaving the cladding material unaffected. After the etching process, a conical shape with a smooth and gradual taper is created [42]. The Turner method involves accurately placing the fiber's tip at the boundary between hydrofluoric acid and the organic overlayer, resulting in the production of a significant taper angle and tip diameter. However, the etching process using the Turner method is highly susceptible to environmental influences [43]. Fig. 8(A–E) displays the preparation methods for etching. These sensors can identify trace levels of harmful compounds or pathogens, enabling rapid, on-site food quality assessments. The integration of these sensors into food packaging can offer continuous monitoring of freshness, extending shelf life and enhancing consumer safety.

#### 2.4.2 Bottom-up methods

The bottom-up process involves adding and rearranging atoms and molecules to shape the nano-sensor. Atomic force microscopy is used to rearrange atoms and molecules, assembling smaller components to give the nano-sensor its desired shape. There are two common bottom-up nano-sensor manufacturing processes: gas-phase and liquid-phase synthesis. The possible techniques for this method include wet synthesis or the decomposition of organic metals, chemical vapor deposition (CVD), the sol-gel process (SGP), laser pyrolysis, plasma arcing (P.A), molecular beam epitaxy (MBE), and self-assembly (MSA).

##### *2.4.2.1* Plasma-arcing *(P.A)*

P.A is a prominent nanoparticle and nano-sensor formation process. Positive ions are deposited as nanoparticles, which requires highly ionized gas atoms with high energy, causing electrons to leave the material's valence shell and resulting in atoms carrying a positive charge. Electron detachment from atoms is feasible due to a substantial potential difference. It utilizes an inert gas for heating, and the formation of an arc between the electrodes is triggered by an electron avalanche. The ions produced in these reactions possess significant amounts of kinetic energy, and the deposition of nanoparticles at the cathode is facilitated by the voltage-driven movement of ions [43]. The plasma-arcing method has shown promising results for creating nano-sensors that detect contaminants like pesticides, spoilage-related compounds, and pathogens. For instance, metal oxide nanoparticles synthesized via plasma deposition have been used in sensors for detecting chemical residues, enhancing food quality control processes [44]. Additionally, plasma-assisted techniques are valued for producing high-purity nanoparticles with controlled size distribution, essential for consistent sensor performance in food safety applications [45].

##### 2.4.2.2 Chemical vapor deposition (CVD)

CVD uses substrates and volatile precursors. Precursors react with the substrate, breaking it down at high temperatures and depositing crystals. This method is frequently used in the semiconductor industry [46]. CVD deposits thin films of diverse materials on substrates by chemically reacting gaseous precursors. In a heated reaction chamber, these precursors react and disintegrate on a substrate to generate a solid layer during CVD. At high temperatures (750 °C), CVD may synthesize multiwalled carbon nanotubes (MWCNTs) from carbon-based precursors like ethanol and an inert carrier gas like argon. Uniform and controlled deposition is necessary for nano-sensor property repeatability [47]. In addition, Plasma enhancement (PECVD) lowers reaction temperature and allows the creation of more sensitive nanostructures with less energy. This version allows deposition at lower temperatures while preserving high-quality material growth, increasing adaptability and industrial scalability [48]. Moreover, CVD-fabricated nano-sensors are highly effective for food safety applications due to their sensitivity and specific surface interactions. For example, MWCNT-based thin films prepared via CVD have been utilized for CO2 detection, which can signal spoilage in food storage. The precise control over the growth conditions ensures the high surface area and uniformity required for detecting low concentrations of gases and volatile organic compounds (VOCs), key indicators of food quality [49].

##### 2.4.2.3 Molecular beam epitaxy (MBE)

MBE is a physical evaporation method that does not include any chemical reactions. The procedure is conducted inside a low-temperature setting and involves vacuum evaporation, where atomic beams are directed onto a heated substrate under high vacuum conditions [50]. Molecular Beam Epitaxy (MBE) is a physical vapor deposition technique that allows for the precise formation of thin films and nanostructures at an atomic level without chemical reactions. The process is performed in an ultra-high vacuum (UHV) environment, typically in the range of 10^−8^ to 10^−11^torr, to prevent contamination and ensure high purity. During MBE, atomic beams of source materials (such as metals or semiconductors) are evaporated and directed onto a heated substrate, where they condense and form an epitaxial layer. The temperature of the substrate is carefully controlled to facilitate crystal growth, resulting in highly uniform and well-defined nanostructures. This method allows for layer-by-layer construction with precise control over thickness and composition. In addition, MBE is ideal for nano-sensors that require clean, regulated materials. The method's ability to manufacture thin films with few faults makes it perfect for constructing sensors that detect low levels of pesticides or food deterioration indications. The sensitive photodetectors and gas sensors made from MBE-grown semiconductor films may identify substances produced by spoilt or tainted food [51].

##### 2.4.2.4 Sol-gel process (SGP)

The sol-gel process involves the amalgamation of sol and gel. A sol consists of solid colloidal particles evenly scattered throughout a liquid, while a gel is a solid-like material formed by linked nanostructures in a liquid phase. This technique involves the blending of solid nanoparticles in a liquid media, resulting in the formation of a gel with a pore diameter in the nanoscale range. The characteristics of sol-gels are influenced by factors such as temperature, catalysts, pH, duration, and solvent type [52]. The procedure involves a series of operations including mixing, sol formation, aging, gelation, drying, dehydration, chemical stabilization, and densification [53].

#### 2.4.3 Molecular self-assembly (MSA)

Molecular self-assembly (MSA) is a highly efficient method for producing nano-sensors. Molecular self-assembly is the process by which natural materials, such as DNA, are produced. This process involves the assembly of atoms and molecules to create a precisely defined nanostructure that is held together by noncovalent bonds. The resulting device has the capability to create nanostructures with dimensions ranging from 1 to 100 nm [54].

### 2.5 Inorganic nano-sensors

#### 2.5.1 Carbon based-nanomaterials

**Fig. 9:**
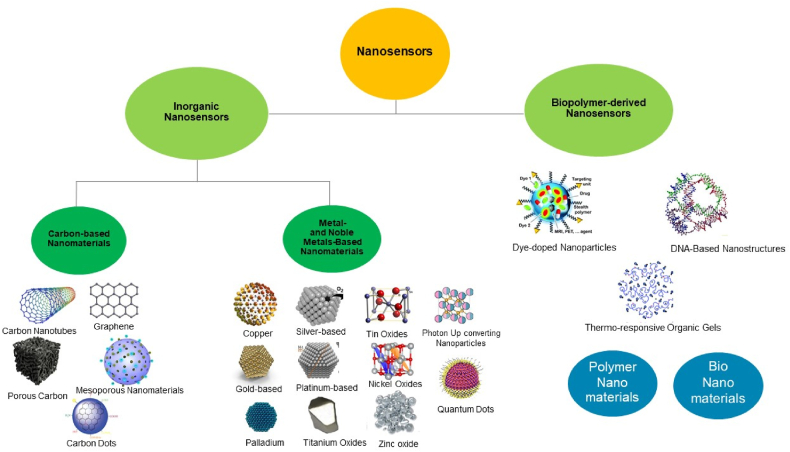
Nano-sensors can be fabricated from various types of nanomaterials.

Carbon-based nanomaterials offer a means to expose a functional surface to analytes, making them highly effective for detecting foreign substances. These materials exhibit high stability, excellent conductivity, and are easily amenable to surface functionalization. Graphene, carbon nanotubes (CNTs), and other materials are used in diverse electroanalytical applications. Fig. 9 illustrates the various categories [55].

#### 2.5.2 Nanomaterials composed of metal and noble metals

Metallic nanoparticles possess distinctive chemical and physical characteristics that render them suitable for diverse applications. Metals such as gold, platinum, silver, cobalt, copper, and rare earth metals exhibit significant potential due to their excellent selectivity and stability [56]. These nanomaterials, which are composed of metals, possess a significant surface/volume ratio, rendering them valuable for utilization in diverse domains such as medicine, catalysis, electrodes, fuel cells, and mechanical actuators. Here, we present an elaboration on certain nanomaterials that are based on metal [57].

### 2.6 Nano-sensors produced from biopolymers

#### 2.6.1 Polymer nanomaterials

Polymeric nanoparticles are commonly employed for the detection of hazardous and chemical pollutants in both gaseous and liquid forms, as well as for health-related applications. Nanocomposites composed of carbon nanotubes (CNT), graphene, metal nanoparticles, or a mixture of these materials exhibit improved electrochemical detection characteristics. The properties of the nanocomposites or nano-sensors are greatly influenced by the combination of filler material and matrix in these composites, impacting selectivity, sensitivity, and biocompatibility. Polymeric nanoparticles are widely utilized in various applications within the healthcare sector, such as medicinal coatings, prostheses, implants, and medical equipment [58].

#### 2.6.2 Bionanomaterials

Biomolecules are structured into nanoparticles in the field of bionanomaterials. The incorporation of nanostructures into biomolecules enhances their analytical capabilities, making them suitable for application as nano-sensors. For example, in one study, multiwalled carbon nanotubes (MWNTs) were synthesized to detect capsaicin using electrochemical methods by employing a nano-bio-composite of L-phenylalanine ammonia-lyase enzyme [59]. Furthermore, in another study, nano-sensors for the identification of Escherichia coli were fabricated through the creation of a self-assembled monolayer (SAM) [60].

#### 2.6.3 Nanostructures made of DNA

DNA, a nucleic acid molecule serving as a repository for genetic information, consists of a phosphate group, a sugar molecule, and a nucleobase containing nitrogen. Its double-stranded structure makes it advantageous for utilization in self-assembly procedures in nano-sensors [61]. DNA possesses significant attributes, functioning as an inflexible polymer when its size is below 50 nm. Its molecules can be separated through a self-assembly mechanism, enabling manufacturing at the nanoscale [62]. Genetic information is encoded through chemical coding mechanisms, and intermolecular interactions between molecules can be readily programmed [63]. These features enable the utilization of DNA for constructing nanostructures in nanomaterials.

#### 2.6.4 Dye-doped nanoparticles

The encapsulation of dyes into nanoparticles is performed to produce nanomaterials that are sensitive to temperature. The precipitation method is used to synthesize temperature sensors at the nano-scale. A homogenous solution is created through the dissolution of a host substance and a probe in an organic solvent. An additional solvent, in which the host material and probe are insoluble, is introduced into the current solution. The probe is enclosed within a structure, and subsequently, the host material solidifies. This approach can be used with a wide range of temperature sensors [64].

#### 2.6.5 Thermo-responsive organic gels

Certain organic gels have the ability to detect temperature within a living organism. Phase transition plays a crucial role in temperature detection. Thermo-responsive poly(N-isopropylacrylamide) undergoes a phase transition in an aqueous solution. Below a temperature of 305K, the polymer undergoes swelling. However, as the temperature increases, it undergoes a transformation, forming nanoparticles with a size of 100 nm. The polymer has the capability to retain specific near-infrared (NIR) probes, enhancing its suitability for in vivo temperature sensing [65].

### 2.7 Fabrication of lab-on-chip (LOC) devices

**Fig. 10:**
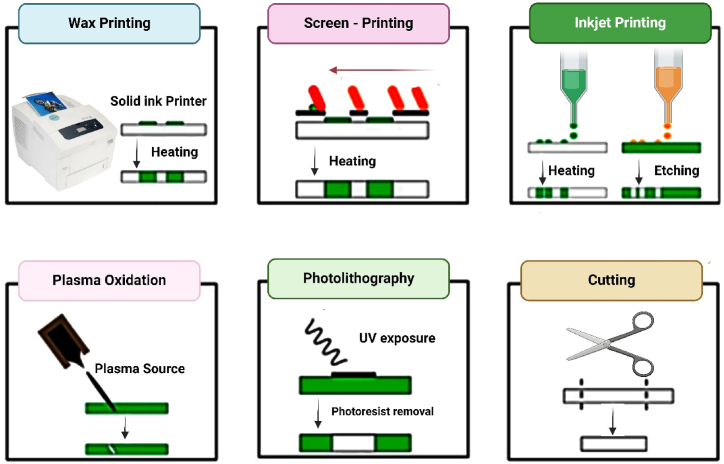
Methods of manufacturing for the construction of lab-on-chip devices.

Lab-on-chip (LOC) technology has become pivotal in advancing food safety and quality monitoring through its innovative fabrication methods and versatile applications. The fabrication of LOC devices typically involves techniques like soft lithography, photolithography, and 3D printing, which allow for the creation of microfluidic platforms capable of performing complex analyses on a compact scale. Soft lithography, for example, uses a polymer like polydimethylsiloxane (PDMS) cast on a master mold to create microchannels for fluid flow, making it an effective and cost-efficient method for LOC device production [66]. More recent advances include the use of 3D printing, particularly Fused Filament Fabrication (FFF), which supports rapid prototyping of customizable LOC devices, expanding their use in various food safety applications through cost-effective production and innovative design flexibility [67].LOC devices integrate essential functions such as sample preparation, detection, and analysis, enabling rapid, on-site testing that significantly reduces the time needed for traditional laboratory-based methods. For example, LOC systems have been shown to effectively detect pathogens like E. coli and Listeria in food samples, combining processes such as cell capture, DNA lysis, and amplification on a single platform. This integrated approach can reduce detection time to mere hours compared to days required by conventional methods, enhancing the responsiveness of food safety protocols [68]. Additionally, LOC devices are increasingly used for detecting chemical residues and pesticides in food products. Hybrid paper-based LOC platforms, for instance, have been designed to screen for toxic substances like carbofuran in produce, demonstrating their practical application in routine safety checks [69].The ability to incorporate sensors capable of detecting pH changes and volatile compounds has made LOC systems essential for monitoring food quality. This capability is crucial for maintaining the freshness of perishable items during transport and storage, as these sensors provide real-time data that can alert supply chain managers to early signs of spoilage [70]. Overall, the portability, minimal sample requirement, and integration with digital devices for easy data analysis have made LOC technology an indispensable tool in modern food safety management, supporting a shift towards faster, more efficient, and decentralized testing approaches. Fig. 10 shows fabrication method LOC.

### 2.8 Application nano-sensors

The utilization of nano-sensors to identify harmful bacteria in the field of food safety and quality is rapidly advancing and has the potential to fundamentally transform methods for ensuring food and beverage safety. Nano-sensors, known for their exceptional sensitivity and specificity, are being employed to detect and identify bacterial pathogens in different food matrices, averting foodborne diseases and outbreaks. These advanced instruments can identify infections at extremely low levels, well in advance of them posing a threat to public health [71].

Nano-sensors employ several processes to detect pathogens, such as optical, electrochemical, and mass-sensitive methods. For instance, some nano-sensors use gold nanoparticles or quantum dots within a biosensor system to visually detect the existence of infections by means of color alterations. Others utilize carbon nanotubes or graphene-based materials to identify bacterial DNA or specific proteins, providing quick and precise detection capacities [72].

The incorporation of nanotechnology in pathogen detection is especially advantageous for the food industry, where it is crucial to guarantee the sterility and quality of products [72]. Nano-sensors have the capability to be employed in food production facilities, packaging, and even integrated into smart packaging to consistently monitor the existence of detrimental bacteria including *Salmonella*, *Listeria*, and *E. coli*. This not only aids in preserving the quality and prolonging the shelf life of food goods but also substantially diminishes the likelihood of foodborne illnesses [73].

#### 2.8.1 Detection of pathogens bacteria in foods

##### 2.8.1.1 E coli (Escherichia coli)

*Escherichia coli*-O157H7 is the most significant strain among all *E. coli* strains due to its ability to produce toxins that can damage the intestines, leading to symptoms such as stomach ache, bloody diarrhea, and Haemolytic–uraemic syndrome (HUS). Even a small amount of 100 cells can cause infection [74]. Human transmission can occur through the consumption of raw or undercooked minced beef and unpasteurized milk. Foods identified as sources of *E. coli* outbreaks include raw hamburger, salami, contaminated apple cider, yogurt, and cheese made from unpasteurized milk. Contamination of meat, vegetables, water, and fruit with fecal matter also contributes to outbreaks [74,75].

**Fig. 11:**
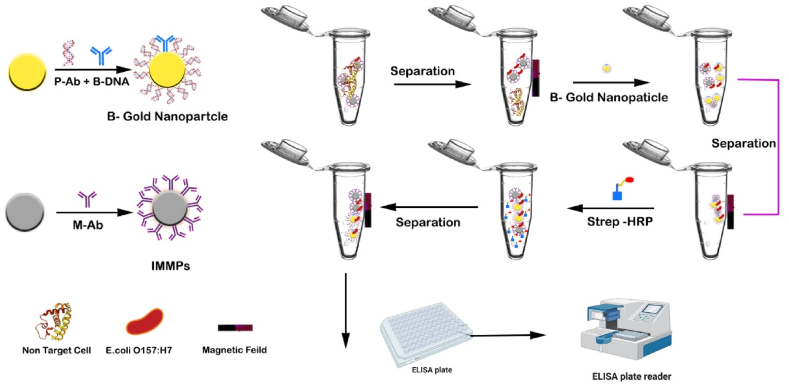
illustrates the process of preparing IMMPs and B-GNPs, as well as the steps involved in separating and conducting immunoreactions with E. coli O157H7 using IMMPs, B-GNPs, and Strep-HRP. E. coli O157H7 detection via the enzyme-linked immunosensor.

In the study developed a DNA sensor based on a quartz crystal microbalance (QCM) for mass enhancers to increase the frequency change using streptavidin-conjugated with MNPs. This sensor is used for the detect of *Escherichia coli-O157H7* by utilizing nanoparticles [76]. A DNA probe, which has been chemically altered with thiol groups, was affixed to the QCM sensor. This probe specifically targets the eae-A gene of *Escherichia coli- O157H7*. Hybridization was initiated by subjecting the single-stranded DNA probe with the complementary target DNA. The process of amplification was carried out using asymmetric PCR, utilizing primers that were labeled with biotin. This is caused to a change in mass, as well as a simultaneous alteration in the frequency of the QCM, which was used to detect *Escherichia coli- O157H7*. The detection limit reached was 267 colony forming units (CFU/mL) within the linear working range of 267 - 267 × 10^4^ CFU/mL [76]. A circulating-flow (PEB) piezo electric biosensor was created to detect *E.coli*- *O157H7* utilizing a Gold Nano Particles-conjugated thiolated probe as a mass intensifier and sequence verifier, following a similar technique [77]. A thiolated probe specific to the eae-A gene of *Escherichia coli*-O157H7, conjugated to piezoelectric biosensor (PEB), was used to detect a gene fragment of *Escherichia coli* amplified by PCR. The resulting change in mass was evaluated as a frequency shift of piezoelectric biosensor. The detection limit achieved was 120 CFU/mL within the linear working range of 100-1,000,000 CFU/mL [77]. A disposable immune-sensing strip was created for *Escherichia coli-O157H7* detection in milk. This strip utilizes a double antibody system for an indirect sandwich enzyme-linked immunoassay. To build the strip, 13-nm gold nanoparticles (GNPs) were attached to screen printe carbon electrodes (SPCEs) [78]. The electrode was connected to the first *Escherichia coli*-*O157H7* specific antibody, *Escherichia coli -O157H7* intact cells, and the second *Escherichia coli-O157H7* specific antibody that was linked to horseradish. The substrate employed was hydrogen peroxide, while the mediator used was ferrocedicarboxylic acid (FeDC). The use of gold nanoparticles (GNPs) and iron dextran-coated (FeDC) particles significantly increased the current responsiveness by a factor of 13.1. This improvement enabled the detection of 6 colony-forming units (CFU) per strip in buffer solution and 50 CFU per strip in milk. This amperometric approach is capable of detecting bacterial various levels of concentration from 102 to 107 CFU/mL. Cho et al. [79]. Fabricated an electrochemical immunosensor by depositing peptide nanotubes on screen-printed carbon electrodes (PNseSPCE). The immobilized anti- *Escherichia coli-O157H7* antibody on PNeSPCE effectively adsorbed *Escherichia coli-O157H7* from samples by means of antigene-antibody interaction. Additional *E. coli* strains were identified with the use of sensors that integrated nanomaterials, including MNPs [80], SNPs [81], GNPs [82], and carbon nanotubes (CNTs). In the study devised a technique to identify *E. coli* cells at a concentration of 10^4^ cells/mL using D-mannose-functionalized magnetic nanoparticles (MNPs). The process involved the modified MNPs incubation with fluorescein-labeled concanavalin A at a temperature of 4 °C for a duration of 12 h. Subsequently, the MNPs were further incubated with *Escherichia coli-O157H7* cells in a (PBS) phosphate buffer saline. The magnetic field was then employed to separate the MNPs, which were subsequently stained with a fluorescent dye and examined using epifluorescent microscopy [80]. In a comparable investigation, Kalele et al. employed rabbit immunoglobulin-G (IgG) antibody-conjugated silver nanoshells to swiftly and extremely selectively identify E. coli within the range of 5-10^9^ cells. This was achieved by observing the alteration in the shift of the surface plasmon resonance (SPR) band in the presence of *E. coli* cells [81]. Furthermore, a swift electrochemical method for detecting *E. coli* was disclosed, utilizing core-shell Cu@GNPs as sensors specifically designed to target *E. coli* Maurer et al. [82]. Despite this, with the help of polyethyleneimine (PEI) coated golden tungsten wire, bionano-sensor single-walled carbon nanotubes (SWCNTs) was developed. The bionano-sensor was able to detection *Escherichia coli* through the activity that occurred at the junction of the golden tungsten and SWCNT wires. The streptavidin and *Escherichia coli*-specific antibody were used to functionalize this junction, and the electrical current change was measured while the material containing *Escherichia coli* was being introduced into the junction. The use of the golden tungsten wire coupled with the SWCNT resulted in an electrical current change of 290.90 near 291 nA, whereas the use of the Single-walled carbon nanotubes SWCNT alone resulted in a change of 33.13 nA in the electrical current. For the developed SWCNT coupled bionano-sensor, the total amount of time required for the detection of *Escherichia coli* was 5 min, and the detection limit was 102 colony-forming units per milliliter [83]. Shen et al. devised a technique known as functional nanoparticle-enhanced ELISA to detect *Escherichia coli-O157H7* in food samples. The magnetic nanoparticles, coated with antibodies specific to *Escherichia coli-O157H7*, were employed to gather and then isolated from the target bacteria using a magnetic separator. Subsequently, polyclonal anti-*Escherichia coli-O157H7* antibodies were applied to Beacon gold nanoparticles (B-GNPs) and then added to the isolated *Escherichia coli-O157H7* complex. This facilitated the occurrence of an immunoreaction. The unbound B-GNPs were subsequently extracted from the complex using a magnetic separator. Afterwards, Strep-HRP was added to the solution to interact with the pre-existing polyclonal anti-*Escherichia coli-O157H7*. Subsequently, any unattached Strep-HRP particles were removed using a magnetic separation technique. The color change, caused by *Escherichia coli-O157H7,* was checked by ELISA test. This is shown in Fig. 11 [84]. A separate investigation demonstrated the use of single-walled carbon nanotubes (SWCNTs) in a nano-sensor, employing the potentiometric approach, can detect 6 coliform units per milliliter in milk and 26 coliform units per milliliter in apple juice within a timeframe of one to 2 min [85]. The specific strain of *Escherichia coli* known as O157H7. On the other hand, Phage-type M13 KE phage nano-sensors, specifically designed for water, orange juice, and skim milk samples, use a fluorescent solution method to detect *Escherichia coli-K12*. These nano-sensors were able to detect 50 coliform units per milliliter in water and 5 coliform units per milliliter in orange juice and skim milk within a time frame of less than 4 h [86]. It has recently fabricated innovative nano-biosensor platforms by attaching RNA-coated GNPs to CNTs. This platform is specifically designed for determination of *Escherichia coli*. Finally, the development of nano-sensors for the detection of *Escherichia coli*, is in progress and nanoscale will reduce the risks of this bacterium. In addition for lab on chip, Guo et al. employed electrochemical impedance spectroscopy to identify *E. coli*, achieving a detection limit of 102 CFU/mL. Once more, these detection limits remain subpar (or the test duration is excessively prolonged) compared to those of "labeled" ELISA lab-on-a-chips [87].

##### 2.8.1.2 Salmonella

*Salmonellosis* is a significant bacterial disease mostly caused by *Salmonella species*, including *S.* Enteritidis and *S.* Typhimurium [88]. According to WHO estimates, there are around tens of millions of new instances of human infection each year, resulting in 100,000 fatalities. Symptoms of this infection include Pyrexia, Abdominal discomfort, gastroenteritis, vomiting, and nausea [74]. In the study demonstrated a highly sensitive electrochemical immune-assay for detecting *Salmonella* Typhimurium. They immobilized mono-clonal antibodies on polystyrene to capture the bacteria. Subsequently, a polyclonal antibody-GNPs conjugate was introduced to facilitate the binding of the bacteria, in the presence of a Cu or Cu-enhancer solution and ascorbic acid. The Cu generated during the reduction process was selectively accumulated onto gold nano particles in order to directly quantify the concentration of *S.* Typhimurium through anodic stripping voltammetry. This method detection limit was 98.9 CFU/mL, and the anodic current showed a linear relationship with the *Salmonella* Typhimurium concentration within the range of 1.30 × 10^2^ to 2.6 × 10^3^ CFU/mL [89]. A further investigation developed a reusable capacitive immunosensor that utilized ethylene-di-amine and gold nanoparticles attached to a glassy carbon electrodes (GCEs). This immune-sensor was designed for *Salmonella* sp. detection in some pork samples [90]. The direct measurement of the interaction between monoclonal *Salmonella species* with antibody GNPs conjugated, can be achieved by using Electrochemical impedance spectroscopy (EIS) with the detection limit 1 × 10^2^ (CFU/mL). Joo et al. for *Salmonella* detection in milk, devised a simple and highly sensitive technique by employing optical nanocrystal probes and Magnetic nanoparticles (MNPs). The milk bacteria were trapped by MNPs-linked antibodies. The bacteria-MNP complexes were separated from the solution by using a magnetic field. Next, the complexes were exposed to TiO_2_ nanocrystals that were immobilized with antibodies, allowing them to absorb UV light. Finally, the MNP-*Salmonellae*-TiO2 complexes were magnetically separated from the solution in order to analyze the TiO2 nanocrystals that were not bound using a UV–visible spectrometer. Although, the Salmonella detection limit in milk was 100 CFU/mL [91]. Jain et al. tried to optimize the efficacy of an electrochemical biosensor by integrating carbon nanotubes (CNTs). The researchers fixed carbon nanotubes (CNTs) that had been modified with monoclonal antibodies onto a glassy carbon electrode. The aforementioned configuration was employed to determine *S.* Typhimurium by examining alterations in charge transfer resistance and impedance via electrochemical impedance spectroscopy. The detection method exhibited a linear response range spanning from 10^−1^ to 10^−6^ of an overnight bacterial culture's serial dilution value. The detection limit was determined to be 1.6 x 10^4^ CFU/mL [92]. Quantum dots (QDs) are becoming more commonly used as fluorescent markers, making them a novel and promising type of fluorescent biosensors. Yang and Li conducted a study to identify *S.* Typhimurium in the wash water of chicken carcasses. The bacteria were separated from the wash water by employing magnetic beads that were coated with antibodies specific to Salmonella. Afterwards, an additional biotin-labeled antibody specific to Salmonella was added, allowing for the interaction between biotin and quantum dots coated with streptavidin. This interaction facilitated the quantification of the intensity of fluorescence. An association was observed between the logarithm of bacterial cell number and the intensity of fluorescence between 10^3^-10^7^ CFU/mL [93]. The lowest detectable concentration was determined to be 103 CFU/mL. Prior to the aforementioned method, Weeks et al., *Salmonella enterica* cells detected at concentrations as low as 25 CFU/mL by (SNC) silicon nitride cantilevers, this was achieved by monitoring the surface bending of the cantilever, which was directly correlated with the quantity of bacteria attached to it [94].On the other hand, Peroxidase-gold nanoparticles have been employed to create aptamer-based sensors, for rapid determination of *Salmonella enterica serovar* in milk samples. This detection method utilizes optical techniques and can get results within 3 h, with a sensitivity of 1 × 10^3^ CFU/mL coliform unit per milliliter [95]. Moreover, an integrated lab-on-a-chip (LoC) platform and process were developed by Tsougeni et al. for quick pathogen analysis in food samples. An oxygen plasma nanotextured polymeric chip in a cohesive microfluidic chamber captures bacteria by immunoaffinity, chemical lysis, and isothermal DNA amplification. The analytical time from sample to result was under 4.5 h, a fivefold improvement over standard procedures. Without labels, *Salmonella, B. cereus, Listeria, and E. coli* can be detected in milk with minimal off-chip processing and fast analysis [96]. Table 2 displays various designed nanoparticles for *Salmonella* germs detection.

**Table 2:** Creation of nano-sensors designed to detect *Salmonella* germs.

Serotype	Nano-sensor Type	Application	Limit of detection	Time
**S. Typhimurium**	Gold nanoparticles–urease	Chicken- duck	102 CFU/mL	120 min
**S. Typhimurium**	nickel nanowires	Chicken	80 CFU/mL	120 min
**S. Typhimurium**	(MWCNT)s	Chicken	80 CFU/mL	10 min
**S. Typhimurium** **S. Enteritidis**	AuNRs	Buffer	23–108 CFU/mL	<1 min
**S. Typhimurium**	Apt/AuNPs@AuNDs	Milk	35 CFU/mL	60 min
**S. Typhimurium**	Melittin/Fe3O4/SPIDE/Abd	Apple juice and potable water	10 CFU/mL	25 min
**Salmonella**	Lab on chip (LOC) Base Immunomagnetic beads	Pork	50 cells per test	40 min
**Salmonella**	Lab on chip (LOC) Base Magnetic bead	MILK	10 cells for each kind of pathogen	15 min
**S. Typhimurium**	Lab on chip type Microfluidic chips	Fresh cut salad	6.1 × 101 CFU/mL	45 min
**S. Typhimurium**	Lab on chip Finger-actuated microfluidic Biosensor	Chicken	14 CFU/Ml	60 min

##### 2.8.1.3 Mycobacterium avium

The paratuberculosis sub-species *Mycobacterium avium* is a pathogen which responsible for Johne's disease in cattle. The main challenge in limiting the transmission of this illness is the difficulty in quickly identifying this germ at low concentrations. Advancements in the development of nano-sensors offer appealing alternatives for rapid, sensitive, and efficient analysis. Kaittanis et al. devised a single-step technique for detecting bacteria in milk and blood using superparamagnetic iron oxide nanoparticles (SPIONs), which takes advantage of the magnetic relaxation characteristic of these nanoparticles. The method of magnetic nano-sensors is based on their capacity to transition between scattered and clustered states when interacting with a target. This transition leads to a simultaneous alteration in the relaxation period of spine spins. To detect MAP, the SPIONs were linked to anti-MAP antibodies via protein G. The nano-sensors demonstrated a dose-dependent response when MAP was added, with the most effective results observed at a nanoparticle concentration of 2 μg Fe per microlitre. As a result, the mean arterial pressure (MAP) increased in whole milk that was mixed with 2 μg of iron per microlitre. The MAP nano-sensor detected a change in T2, it was inversely proportional to the MAP concentration. The accurate measurement was achieved within the range of 15.5–775 coli form unit per milliliter after a 30 min incubation at 25 °C. Nevertheless, at a temperature of 37 °C, the identification and measurement of MAP could be accomplished with great accuracy in 2 % milk. An additional benefit is that exposing the sample to a (30 min) incubation at 37 °C did not impact the ability to detect another bacteria, such as (*Escherichia coli, Staphylococcus aureus, Enterococcus faecali, Proteus vulgaris, Pseudomonas aeruginosa, and S.marcescens)*. However, extending the incubation time to 45 min resulted in an increase in the minimum detectable concentration from 15.5 Colony forming units to 38.8 Colony forming units. In addition, this assay has the capability to ascertain the MAP status (positive or negative) of blood samples from an individual in a single step [94,97]. In a separate investigation, Yakes et al. devised a sandwich immunoassay to swiftly and accurately detect Mycobacterium avium subsp. paratuberculosis (MAP) at low concentrations. This method utilized (SERS) surface enhanced Raman scattering and involved two crucial elements: the immobilization of 13E1, monoclonal antibody, to target the MAP2121c which is a surface protein on the microorganism, and the creation of extrinsic Raman labels using 60 nm gold nanoparticles to selectively bind the captured proteins and generate strong SERS signals. Li and Church have conducted a review on the utilization of Raman spectroscopy for the examination of nanomaterials in the fields of food and pharmaceuticals [98].The creation of the Raman label relied on the natural absorption of sulfur compounds onto gold nanoparticles, followed by the construction of a 5,50-dithiobis(succinimidyl-2 nitrobenzoate) (DSNB) adlayer on the nanoparticle's surface. This adlayer can then attach antibodies, leading to the generation of a biospecific label. The detection relied on measuring the intensity of the powerful ns(NO2) of the DSNB-derived monolayer. The Raman label-incorporated surface-enhanced Raman scattering (SERS)-based immunoassay successfully detected *Mycobacterium avium* subsp. paratuberculosis (MAP) within 24 h. The test achieved a detection limit of 100 ng/mL in phosphate-buffered saline (PBS) and 200 ng/mL in pasteurized whole milk. This was achieved by using a recognition element 13E1 in the SERS platform. The high reproducibility seen in this process can be attributed to the creation of homogenous nanoparticles and the optimization of Raman labels during manufacture [99].

##### 2.8.1.4 Listeria monocytogenes

*Listeria monocytogenes*, Gram-positive bacteria, is the source of the infectious disease listeriosis. Of all the bacterial infections spread through food, it is the most aggressive and the third most deadly. By employing a high-transition temperature superconducting quantum interference device (SQUID) to measure the rate at which antibody-bound magnetic nanoparticles adhere to bacteria, Grossman et al. developed an inventive method for detecting L. monocytogenes. A 50 nm-sized sample of superparamagnetic nanoparticles coated with antibodies was mixed with the Listeria monocytogenes sample. The magnetic dipole moments were then oriented using a pulsed magnetic field.SQUID can be used to measure the slow release of magnetic flux caused by Néel relaxation in nanoparticles attached to Listeria monocytogenes, as opposed to the rapid randomization of unattached nanoparticles caused by Brownian rotation. In a 20 mL sample volume, the detection limit for Listeria monocytogenes was found to be 560 cells. In addition, 230 cells of Listeria monocytogenes were detected in a 1 nL sample volume*.* [100]. The upper section describes the use of a comparable magnetic relaxation technique for the detection of mycobacterial species. Table 3 shown summarize nanosensors application in food and detection listeria monocytogenes.

**Table 3:** Nano-sensors designed to detect *Listeria monocytogenes* in different types of food.

Type of Food	Nano-sensor Type	Detection Limit	Reference
Whole milk, ground meat	Magnetic nanoparticle-based colorimetric assay	2.17 × 10^2^ CFU/mL	[101]
Lettuce, milk, ground beef	Impedance immunosensor with magnetic nanoparticles and microfluidic chip	10³ CFU/mL	[102]
Spiked food samples	Multicolorimetric assay based on etching of gold nanorods	10 CFU/mL	[103]
Lettuce homogenate	Single-walled carbon nanotubes-based electrochemical impedance immunosensor	10³ CFU/mL	[104]
Spiked food samples	Nanohybrid quantum dot complex	5.19 × 10³ CFU/mL	[105]
Artificial contaminated food	Gold nanoparticle-based colorimetric detection with hyperbranching rolling circle amplification (HRCA)	75 copies	[106]
Lettuce, tomato, ground beef	Magnetic nano-beads based separation combined with propidium monoazide treatment and multiplex PCR	5.4 × 10^2^ CFU/g	[107]
Meat samples	Optical label-free biosensor	Not specified	[108]
Ready-to-eat meat samples	Multiplex fiber optic biosensor	10³ CFU/mL	[109]
Spiked food samples	Nanoporous sensor with aptamer	100 CFU/mL	[110]

##### 2.8.1.5 Pseudomonas aeruginosa

**Fig. 12:**
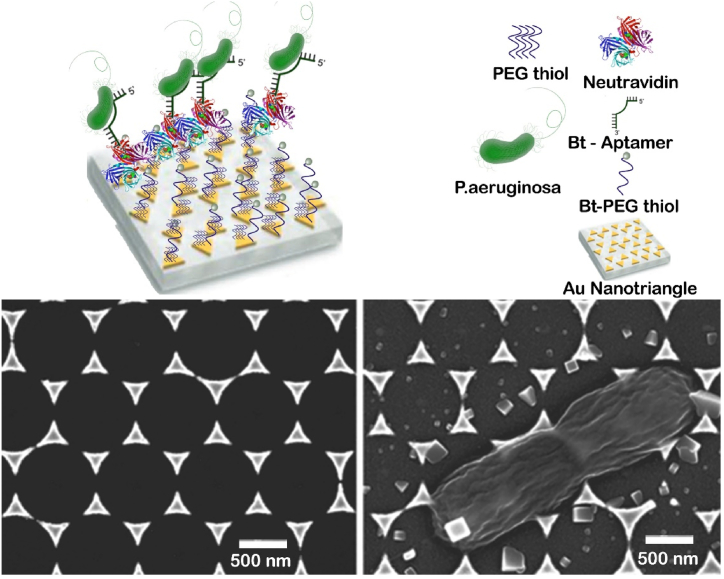
illustrates the operational mechanism and detection process of *Pseudomonas* bacteria for the nanosensor.

*Pseudomonas aeruginosa*, a ubiquitous Gram-negative bacterium, is renowned for its capacity to induce inflammation and sepsis. Importantly, the invasion of specific organs such as lungs, urinary system, and kidney can result in life-threatening outcomes. Moreover, it is accountable for nosocomial infections in healthcare facilities and medical apparatus, such as catheters. Norman et al. showed a study on the targeted elimination of *Pseudomonas aeruginosa* using gold nanorods. The gold nanorods, which have amine terminations, were covalently attached to carboxylic acids obtained from primary antibodies against *P. aeruginosa.* This attachment was achieved using 1-ethyl-3-(3-dimethylaminopropyl)carbodiimide, a compound commonly used in a chemical process called carbodiimide chemistry. Subsequently, the mixture consisting of antibodies, nanorods, and bacteria was subjected to near-infrared (NIR) light with a wavelength of 785 nm and a power of 50 mW for a period of 10 min. Afterwards, the suspension was dyed with vital (green) and non-vital (red) dyes, and the quantity of living and non-living cells was assessed. The cell viability of P.aeruginosa cells coated with nanorods and exposed to NIR radiation decreased by 75 %, compared to the 80 % cell viability observed in both NIR-exposed cells with and without nanorods that were not exposed to NIR [111].on the other hand A study was conducted using Au-N triangles nanoparticles to detect the presence of P. aeruginosa bacteria in water samples. This was achieved by utilizing the localized surface plasmon resonance signal. Fig. 12 below shows the imaging of a single bacterial cell using scanning electron microscopy (SEM) [112].

##### 2.8.1.6 Bacillus cereus

**Fig. 13:**
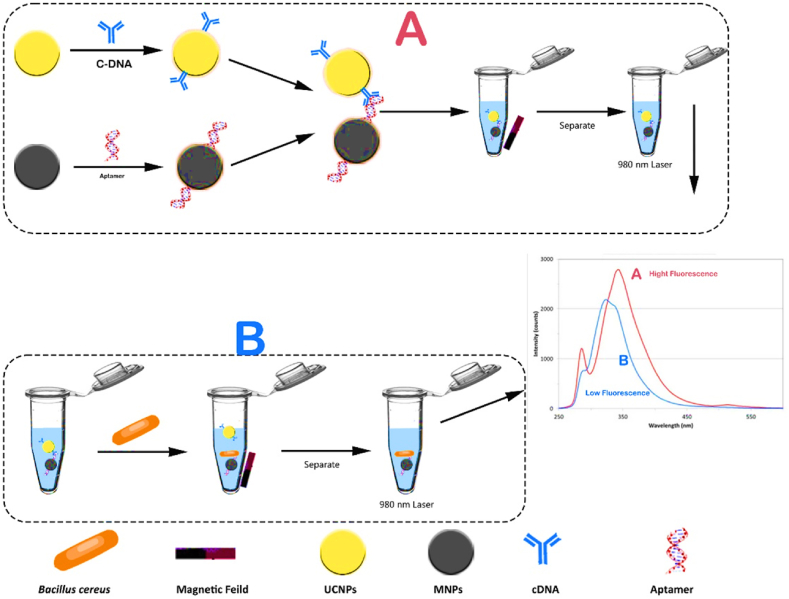
steps involved in detecting the presence of *bacillus*.

**Fig. 14:**
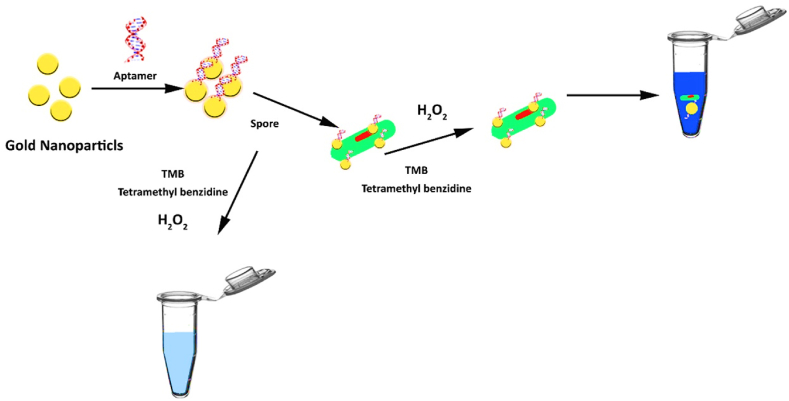
The colorimetric assay scheme is designed to detect *B.cytotoxicus* spores by utilizing the spore-enhanced peroxidase-like catalytic activity of gold nanoparticles.

*Bacillus cereus*, a gram-positive bacterium that forms spores, is widely recognized as the primary culprit behind foodborne illnesses. This bacterium is mostly found in soil and is commonly found in plant-based foods. However, because to its widespread presence and the ability of its endospores to withstand harsh physical conditions, it can also be found in other forms of food such as meat, eggs, dairy products, and processed foods. Furthermore, the emetic form is triggered by the ingestion of food contaminated with cereulide toxin, which is synthesized by bacteria. As a result, individuals experience symptoms of emesis and queasiness. The second type is caused by enterotoxins produced by B. cereus in the small intestine, leading to symptoms such as diarrhea and abdominal pain [113]. A study was conducted to assess the ability of Bacillus to cause disease. In this study, aptamers (Apt) were attached to the surface of magnetic nanoparticles (MNPs) to create Apt-MNPs capture probes. The results of the study demonstrated that this method has a strong ability to accurately quantify B. cereus within a range of 48–49 × 10^6^ CFU/mL, under optimal conditions. The detection limit of this method was found to be 22 CFU/mL. Furthermore, the proposed detection method also demonstrates a high level of specificity. Fig. 13 depicts the sequential steps involved in detecting the presence of *bacillus* [114]. In addition, the suggested detection method also exhibits a notable degree of specificity. In addition, GNPs were utilized in a distinct investigation to detect *bacillus* infections in water, milk, and cooked potatoes. The experiment was assessed through both visual observation and quantitative analysis using a spectrophotometer. The BAS6R@MPs aptasensor, when used with BAS6R@AuNPs, demonstrated exceptional sensitivity, enabling the detection of bacterial concentrations as low as 10^2^ CFU/mL in water and milk, and 10^4^ CFU/mL in mashed potatoes, visible to the naked eye. Furthermore, the researchers effectively differentiated between spores of *Bacillus cytotoxicus* and *Bacillus subtilis*, as well as bacterial vegetative cells, in contaminated food samples. This demonstrates a significant degree of selectivity. Fig. 14 illustrates the sequential process of identifying the existence of bacillus [115].

##### 2.8.1.7 Shigella

*Shigella* is a collection of bacteria that possess specific traits: they are Gram-negative, capable of surviving with or without oxygen, do not form spores, lack the ability to move, and have a cylindrical shape. Furthermore, *Shigella* is genetically closely associated with *Escherichia*. The medical term "shigellosis" is commonly used interchangeably with the term "bacillary dysentery." The onset of diarrhea, which may manifest as either watery or bloody stools, along with fever and abdominal cramps, usually takes place within a period of 1–2 days following the ingestion of the bacteria. This condition typically resolves within a period of 5–7 days. In study presents a new nanoplatform consisting of biofunctionalized magnetic nanoparticles (MNPs) that have been modified with upconversion nanoparticles (UCNPs). The primary objective of this nanoplatform is to rapidly and precisely identify the existence of *Shigella*. The MNPs@UCNPs fluorescence biosensor effectively identified the existence of *Shigella* within a 1-h period, with a minimum detectable concentration of 32 colony-forming units per milliliter (CFU/mL). The study showcased a rapid and specific sensing platform that produced outstanding results during the analysis of chicken samples [116]. *Shigella* bacteria were detected in milk and chicken breast samples during a separate investigation. A composite material was created by combining the Raman active 4-MBA ligand of the Eu-complex with citrate-stabilized Au nanoparticles (cit-Au NPs). This material had two functions, acting as both a reactive base and a Raman indicator. Specific aptamers that selectively bind to *S. Sonnei* were immobilized onto the surface of this material with dual functionality. The *Shigella species*, such as *S. dysenteriae, S. flexneri, and S. boydii*, exhibited a remarkable level of specificity. Through the implementation of experiments on authentic samples, the developed technique exhibits significant promise in producing a diverse array of aptasensors capable of efficiently and conveniently detecting various food hazards [117].

##### 2.8.1.8 Staphylococcus aureus

*Staphylococcus aureus* is a coccus-shaped bacterium that is Gram-positive and classified within the *Bacillota phylum*. It is frequently present in the body's microbiota, specifically in the upper respiratory tract and on the skin. Common symptoms include the abrupt onset of vomiting and stomach discomfort. A novel colorimetric test for detecting *S.aureus* has been developed employing gold nanoparticles and aptamers, combined with TSA for enhanced identification. The devised approach has a detection sensitivity of 9 colony-forming units per milliliter (CFU/mL) and a wide linear range from 10 to 10^6^ CFU/mL [118]. Furthermore, this established technique was effectively employed for the analysis of milk samples. Nano-sensors have been created to detect *Staphylococcus aureus* in food [119]. A nanobiosensor was developed using paper and DNA-Au/Pt bimetallic nanoclusters. This nanobiosensor has a detection limit of 80 CFU/mL [120]. Designed a PDMS microfluidic impedance-based immunoassay sensor with a high sensitivity of 10^2^ CFU/mL. In Sung's study conducted in 2013, antibody/AuNPs/magnetic nanoparticle nanocomposites were employed for immunomagnetic separation and colorimetric detection. The detection limits for *S. aureus* in PBS and milk samples were determined to be 1.5 × 10^3^ and 1.5 × 10^5^ CFU, respectively [121]. In a recent study, examined the application of aptasensors in detecting *S. aureus*. The author emphasized the promising prospects of integrating aptasensors with nanomaterials. These studies collectively show that nano-sensors have the ability to quickly and accurately detect S. aureus in food [122]. Another method involves an aptamer-quantum dot and teicoplanin-gold nanoparticle-based fluorescence resonance energy transfer (FRET) sensor. This sensor offers a detection limit of 2 CFU/mL for S. aureus in buffer solutions and 100 CFU/mL in food samples like milk and orange juice, demonstrating high specificity and sensitivity [123].A dual electrochemical/colorimetric magnetic nanoparticle/peptide-based platform has also been developed. This sensor uses magnetic nanoparticles linked with specific peptides that, upon cleavage by S. aureus protease, reveal a color change detectable by the naked eye and an electrochemical signal proportional to the bacterial concentration. This platform can detect *S. aureus* at levels as low as 3 CFU/mL within 1 min [124].Moreover, a novel approach using upconversion nanoprobes regulated by horseradish peroxidase for dual-mode detection has been created. This method combines aptamer-labeled magnetic nanoparticles and horseradish peroxidase-functionalized upconversion nanoparticles, achieving detection limits of 22 CFU/mL for fluorescence and 20 CFU/mL for colorimetry in meat samples [125].

#### 2.8.2 Detection of toxic bacteria in foods

##### 2.8.2.1 Cholera toxin (CT)

Bacterium *Vibrio cholera* released a complex protein named Cholera toxin, which is responsible for watery diarrhea in individuals infected with cholera. The structure of this complex is oligomeric, specifically defined as AB5, and consists of six protein subunits one A, five B subunits) [74,126]. Viswanathan et al. utilized recent advancements in nanoparticle technology to create a highly sensitive method for detecting CT. They developed sensor that employed liposomal magnification and poly(3,4 ethylene-di-oxy-thiophene) coated on a Nafion-supported (MWCNTs) film on a glassy carbon electrode linked with anti-Cholera T-B subunit monoclonal antibody. The detection method relied on electronic transducers used sandwich-type assay. In this assay, the toxin is initially attached to the anti-cholera Toxin antibody and then with conjugated ganglioside-functionalized liposome. The inorganic compound (C₆FeK₄N₆) Potassium ferrocyanide molecules liberated from liposomes and linked to the electrode were quantified by adsorptive wave square stripping voltammetry. The limit of detection and the linear range of cholera toxin were 10^−16^ g/ml - 10^−14^ - 10^−7^ g/ml [127], respectively. In a separate investigation, gold nanoparticles (GNPs) were attached to a lipid bilayer that contained gangliosides in order to detect CT [128]. This approach offers a significant enhancement in sensitivity, with a 100-fold improvement compared to other standard fluorescent immune-assays (5 nM). The detection limit is 10–100pM and the linear dynamic range spans from 10pM to 100 nM. Schofield et al. devised a colorimetric bioassay where a thiolated-lactose derivative formed self-assembled structures on 16 nm gold nanoparticles (GNPs) [128]. These structures aggregated when they bound to the CT-B subunit, resulting in a color change from red to purple, which served as the basis for detection. The estimated limit of detection was 3 mg/mL [129].

##### 2.8.2.2 Staphylococcal enterotoxin

*Staphylococcal enterotoxins*, a significant group of twenty-one thermally stable toxins produced by *Staphylococcus aureus*, are associated with foodborne illnesses caused by consuming spoiled foods.Exposure to Staphylococcal enterotoxins, even at a low concentration of 20–100 ng per person, causes anorexia, nausea, vomiting, and diarrhea, which are indicative of food poisoning [130]. Moreover, SEs have been associated with the emergence of conditions such as atopic eczema, rheumatoid arthritis, and toxic shock syndrome [131,132]. Although ELISA and other immunological assays are known for their speed and high throughput, they do not possess sufficient sensitivity for specific applications. To overcome this constraint, Yang et al. developed an optical immunosensor that employs carbon nanotubes (CNTs) for the detection of Staphylococcal enterotoxins. This is accomplished by interacting the carbon nanotubes (CNTs) with a fixed anti-SE primary antibody, and then attaching a secondary antibody labeled with horseradish peroxidase (HRP). Subsequently, the fluorescence of horseradish peroxidase is employed for the purpose of identifying the existence of Staphylococcal enterotoxins. The sandwich immunosensor-based assay provides a signal that is six to eight times greater than the standard immunosensor. The detection limit of this is 0.1 ng/mL and it has a linear dynamic range of 0.1–100 ng/mL. However, when applying this test to real food samples such as apple juice, soy milk, meat, and baby food, an additional purification step was required using carboxymethyl cellulose chromatography [131]. Afterwards, the research team conducted a study to identify and detect harmful substances in food, specifically focusing on SEs. Then employed gold nanoparticles (GNPs). The gold nanoparticle surface was attached to an anti-SE primary antibody through physical adsorption, and the antibody-GNPs conjugate was attached to a polycarbonate surface. The sandwich ELISA assay was implemented using a secondary antibody (HRP-conjugated antirabbit IgG) to improve chemiluminescence detection. Subsequently, the efficacy of the enhanced chemiluminescence (ECL) immunosensor was evaluated [131]. The approach's limit of detection was determined to be 0.01 ng/mL, which is tenfold more sensitive than both the classic ELISA method and the previously disclosed CNT-based immunosensor test. Gold nanoparticles are not only less poisonous than carbon nanotube, but they also do not require shortening and acid functionalization. As a result, the fabrication of an immunosensor based on GNPs is significantly simplified [131,132]. In addition for Lab on chip Yang et al. integrated carbon nanotubes, increased chemiluminescence, and a cooled charge-coupled device (CCD) detector to augment the detection of *Staphylococcal enterotoxin* B (SEB) in food. Anti-SEB primary antibodies were affixed to the CNT surface, and the antibody-nanotube composite was adhered to a polycarbonate surface. SEB was then identified using an ELISA assay on the CNT-polycarbonate surface, coupled with an improved chemiluminescence assay. SEB in buffer, soy milk, apple juice, and meat infant food was analysed with a detection limit of 0.01 ng/mL utilizing the CCD detector, which exhibited more sensitivity than the traditional ELISA. They also created a lab-on-a-chip utilizing this CNT-ECL immunoassay to identify SEB [133].

##### 2.8.2.3 Shiga toxin

Shiga-like toxins, which are part of the alike family as the Cholera T toxin, are synthesized by *Escherichia coli* bacteria, particularly the foodborne pathogen *Escherichia coli-O157H7*. The subunit B of shiga same toxin produced by *Escherichia coli-O157H7* has a specific affinity for the globotriose (P^k^) blood group antigen. This antigen consists of the trisaccharide α-Gal(1 → 4)β-Gal(1 → 4)β-Glc. Each of the 5 B subunits of the toxin has 3 binding sites that can interact with the P^k^ antigen [126,134]. Chien and colleagues utilized this phenomena to create an SPR competition assay. They achieved this by self-assembling two variants of P^k^ onto glyconanoparticles of varying sizes 4 nm, thirteen nano meter, and twenty nano meter made of GNPs. Increasing the length of the chain was found to improve the binding strength of the P^k^ moiety, leading to increased flexibility of the P^k^ ligand to attach to several places on the toxin surface. Similarly, the P^k^eg old variants made with larger diameter GNPs exhibited a higher binding affinity, which can be attributed to the reduced curvature of the GNPs. A chip-based assay was designed by including glyconanoparticles, based on the results obtained [134]. Nagy et colleagues conducted a subsequent investigation where they enhanced a chromatic sensor using (Gal-α1,4-Gal) glycol-polydiacetylene nanoparticles to specifically detect Shiga toxin-producing *Escherichia coli-O157H7*. The plates containing shiga toxin producing *Escherichia coli-O157H7* exhibited a color transformation from purple to brown within a span of 5 min. In contrast, the non-Shiga toxin generating *Escherichia coli* solution maintained its purple hue after the addition of glycol-polydiacetylene nanoparticles. This approach demonstrated great selectivity, speed, and sensitivity, with a limit of detect of 1200 U/ml a linear range changed of 1200–7200 U/ml [135].

## 3 Challenges and future strategies for nano-sensors

Nano-sensors represent a promising technology for advancing food safety, environmental monitoring, and healthcare, but significant challenges remain that must be addressed to realize their full potential. The cost of production and scalability issues hinder their widespread deployment. Sophisticated materials like gold nanoparticles and carbon nanotubes are essential for performance but increase production costs, limiting accessibility in resource-constrained environments [17]. To mitigate these challenges, research into cost-effective alternatives and additive manufacturing methods can reduce material consumption and simplify fabrication processes [136]. Furthermore, environmental sensitivity is a double-edged sword; while it allows for high precision, it also exposes these sensors to potential degradation under varying conditions such as humidity or temperature. Future strategies should focus on developing more robust coatings and packaging solutions that maintain sensor stability without compromising performance in diverse environments [137].Additionally, regulatory challenges create barriers to commercial adoption, as nano-sensors involve materials with uncertain long-term environmental and health impacts. Many countries lack clear guidelines for approving nanotechnology products, which discourages investment and slows market entry. Developing internationally harmonized regulatory frameworks will be essential for fostering innovation and enabling companies to invest confidently in nano-sensor technologies [138]. Moreover, scalability remains a pressing issue. Current fabrication techniques, such as chemical vapor deposition and lithography, are not easily adaptable for mass production. To address this, research into new manufacturing technologies, such as printed electronics and nanomaterial-based additive manufacturing, will be crucial in achieving industrial scalability and maintaining reproducibility [139].Furthermore, the development of lab-on-chip platforms and integrated sensing systems offers promising solutions for addressing some of these challenges by combining different sensing technologies into compact, multi-functional devices. Such systems can provide enhanced detection capabilities while reducing overall costs through miniaturization. Future research should focus on modular designs that facilitate easy customization for various applications, from point-of-care diagnostics to real-time environmental monitoring. Collaborative efforts between industry and academia will also play a pivotal role in accelerating the adoption of nano-sensors. Establishing partnerships can bridge the gap between laboratory innovation and market-ready products, ensuring that future developments are aligned with both regulatory standards and commercial needs. Finally, research into eco-friendly materials and sustainable production methods will be essential for aligning nano-sensors with the growing demand for green technologies and minimizing their environmental footprint. With targeted advancements in these areas, nano-sensors can overcome their current limitations and become indispensable tools for the future of smart monitoring and quality assurance across industries.

## 4 Conclusion

In summary, the development of nano-sensor technology represents a significant advancement in the battle against foodborne pathogens. This analysis highlights the exceptional sensitivity, specificity, and rapid detection capabilities of nano-sensors, distinguishing them from traditional detection methods. Their incorporation into food safety protocols offers the potential to greatly improve our ability to monitor and manage foodborne pathogens, ultimately reducing the occurrence of food-related illnesses. Nevertheless, there are still challenges to be addressed, such as refining the technology, ensuring cost-effectiveness, and meeting regulatory requirements. Future research should concentrate on overcoming these obstacles and advancing nano-sensor technology to its maximum potential. The implications of these advancements go beyond food safety, offering broader benefits for public health and environmental monitoring. The ongoing innovation in nano-sensor technology is crucial for creating a safer and healthier future for food consumption.

## CRediT authorship contribution statement

**Farhang Hameed Awlqadr:** Writing – review & editing, Writing – original draft, Software, Investigation, Data curation, Conceptualization. **Ammar B. Altemimi:** Writing – review & editing, Writing – original draft, Software, Investigation, Data curation, Conceptualization. **Syamand Ahmed Qadir:** Writing – review & editing, Writing – original draft, Software, Investigation, Data curation, Conceptualization. **Tablo Azad Hama Salih:** Writing – review & editing, Writing – original draft, Software, Investigation, Data curation, Conceptualization. **Zina T. Alkanan:** Writing – review & editing, Writing – original draft, Software, Investigation, Data curation, Conceptualization. **Qausar Hamed AlKaisy:** Writing – review & editing, Writing – original draft, Software, Investigation, Data curation, Conceptualization. **Othman Abdulrahman Mohammed:** Writing – review & editing, Writing – original draft, Software, Investigation, Data curation, Conceptualization. **Mohammad Ali Hesarinejad:** Writing – review & editing, Writing – original draft, Software, Methodology, Data curation, Conceptualization.

## Consent for publication

All authors approved the manuscript for publication.

## Availability of data and material

All data relevant to the study are included in the article.

## Ethical approval

This article does not contain any studies with human or animal subjects.

## Funding

Not applicable.

## Declaration of competing interest

The authors declare that they have no known competing financial interests or personal relationships that could have appeared to influence the work reported in this paper.
